# Effect of the Lectin of *Bauhinia variegata* and Its Recombinant Isoform on Surgically Induced Skin Wounds in a Murine Model

**DOI:** 10.3390/molecules16119298

**Published:** 2011-11-07

**Authors:** Luiz Gonzaga do Nascimento Neto, Luciano da Silva Pinto, Rafaela Mesquita Bastos, Francisco Flávio Vasconcelos Evaristo, Mayron Alves de Vasconcelos, Victor Alves Carneiro, Francisco Vassiliepe Sousa Arruda, Ana Lúcia Figueiredo Porto, Rodrigo Bainy Leal, Valdemiro Amaro da Silva Júnior, Benildo Sousa Cavada, Edson Holanda Teixeira

**Affiliations:** 1 Integrated Laboratory of Biomolecules (LIBS), School of Medicine of the Federal University of Ceará, Sobral, Ceará 62042-280, Brazil; E-Mails: ziullec@gmail.com (L.G.N.N.); rafaelabastos.ufc@gmail.com (R.M.B.); ffvebio@gmail.com (F.F.V.E.); victorcarneiro@ufc.br (V.A.C.); vassiliepe@gmail.com (F.V.S.A.); 2 Center for Technological Development (CDTec), Unidad of Biotecnology, Federal University of Pelotas, Pelotas, Rio Grande do Sul, 96010-900, Brazil; E-Mail: ls_pinto@hotmail.com (L.d.S.P.); 3 Department of Animal Morphology and Physiology, Federal Rural University of Pernambuco, Recife, Pernambuco 52171-900, Brazil; E-Mails: analuporto@yahoo.com.br (A.L.F.P.); valdemiroamaro@gmail.com (V.A.d.S.J.); 4 Department of Biochemistry and Molecular Biology, Federal University of Ceará, Fortaleza, Ceará 60451-970, Brazil; E-Mails: mayronvasconcelos@gmail.com (M.A.V.); bscavada@gmail.com (B.S.C.); 5 Department of Biochemistry, CBB, Federal University of Santa Catarina, Santa Catarina 88040-970, Brazil; E-Mail: rbleal@gmail.com (B.B.L.)

**Keywords:** wound healing, legume lectins, Caesalpinoideae, *Bauhinia variegata*

## Abstract

Lectins are a structurally heterogeneous group of highly specific carbohydrate-binding proteins. Due to their great biotechnological potential, lectins are widely used in biomedical research. The purpose of the present study was to evaluate the healing potential of the lectin of *Bauhinia variegata* (nBVL) and its recombinant isoform (rBVL-1). Following surgical creation of dorsal skin wounds, seven groups of mice were submitted to topical treatment for 12 days with lectin, d-galactose, BSA and saline. The animals were anesthetized and euthanized on POD 2, 7 and 12 in order to evaluate the healing potential of each treatment. The parameters considered included wound size, contraction rate, epithelialization rate and histopathological findings. Wound closure was fastest in animals treated with rBVL-1 (POD 7). nBVL was more effective than the controls. All skin layers were reconstructed and keratin deposition increased. Our findings indicate that the lectin of *Bauhinia variegata* possesses pro-healing properties and may be employed in the treatment of acute skin wounds.

## 1. Introduction

Skin wounds are the result of disruption of tissue integrity [1]. Tissue damage initiates a cascade of events including inflammation and tissue formation and reshuffle (granulation tissue), eventually leading to partial or complete reconstruction of the damaged area [2,3]. Thus, if a wound is a disruption of anatomical and physiological continuity of an organ or tissue, a scar is an attempt of the body to restore integrity [4].

Skin healing is understood as a dynamic process with a complex cascade of cellular and molecular events involving the extracellular matrix (ECM) and soluble mediators such as cytokines [5]. The repair process starts immediately after injury and includes the following phases: Hemostasis, inflammation, proliferation, ECM remodeling and scar formation (maturation) [6].

A major health concern for many patients, delayed wound healing can significantly reduce quality of life [1,7]. Consequently, the ability of natural products to speed up the healing process with minimal pain and scar tissue formation has been extensively investigated for decades [8].

Lectins are proteins that recognize and bind specifically to epitopes of structural carbohydrates without modifying them [9]. Lectins are mediators in various biological processes, including cell-cell adhesion of fungi and bacteria to host cells, and have long been employed in the detection and analysis of carbohydrates, immune response and other processes [10,11,12]. Due to the participation of lectins in a range of important biological processes, the possibility of using them as biotechnological tools has been extensively explored [13,14,15,16,17,18,19].

Galactose-specific lectins of the subfamily Caesalpinoideae, such as *Bauhinia purpurea* (BPA), *Bauhinia monandra* (BmoLL) and *Griffonia simplicifolia*, have been used as insecticidal agents, as blood group markers, in studies on cancer cells and in several other important biological contexts [20,21,22].

The lectin of the orchid tree *Bauhinia variegata* (nBVL) has at least two isoforms (BVL-1 and BVL-2). Both are galactose ligands with a molecular mass of 32 kDa and structurally similar to other Caesalpinoideae lectins [23]. Alencar and colleagues [24] have shown that nBVL has pro-inflammatory properties capable of inducing resident mast cell-dependent neutrophil migration *in vivo* and *in vitro*.

Plant lectins may also be synthesized with the aid of expression vectors such as bacteria and yeasts, making large-scale production of biotechnically useful lectins feasible [25,26,27]. Recently, through cloning and expression in *E. coli*, a recombinant isoform of the lectin of *B. variegata* (rBVL-1) was obtained, with DNA sequence and amino acids similar to other well-known Caesalpinoideae lectins [23]. The purpose of this study was to investigate the healing potential of topical administration of the lectin of *B. variegata* (nBVL) and its recombinant isoform (rBVL-1) on surgically induced skin wounds in a murine model.

## 2. Results and Discussion

**Figure 1:**
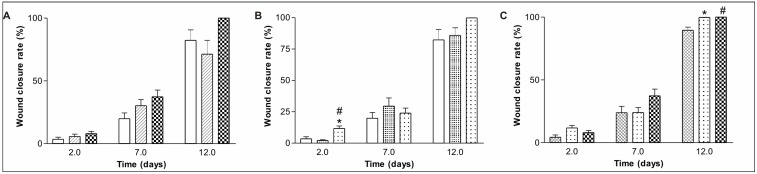
Closure rate of surgically induced skin wounds in mice treated with topical administration of saline 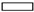; nBVL complexed with D-Gal 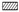 and nBVL 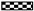 (**A**); saline 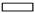; rBVL-1 complexed with D-Gal 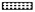 and rBVL-1 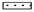 (**B**); * significant in relation to saline; ^#^ significant in relation to rBVL-1 complexed with D-Gal. BSA 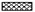; rBVL-1 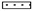 and nBVL 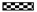 (**C**); * and ^#^ significant in relation to BSA. Statistical test: Mann-Whitney (*p* < 0.05).

Animals treated with rBVL-1 displayed lower levels of typical inflammatory signs, such as swelling and redness, in the first days following treatment, compared to controls, indicating a possible anti-inflammatory effect. In contrast, nBVL had a pro-inflammatory effect, as shown by the finding of higher levels of swelling and redness in the wound bed. On postoperative day (POD) 9, scar tissue was observed in more animals in the rBVL-1 group than in the nBVL group (data not shown). In the inflammatory stage, healing was faster and more effective in G-I (rBVL-1) than in G-VI (nBVL) and controls. Total wound closure was observed in the animals treated with nBVL only on POD 12 (Figure 1A–C).

The measurement of the actual wound area was hampered by the presence of a thick scab (dry fibrin in the wound bed) formed through contact with atmospheric oxygen [28]. Despite this difficulty, reliable measurements were obtained based on the wound borders. Wound epithelization (ER) was significantly greater in G-I (rBVL-1) than in all other groups due to more efficient wound contraction (CR) during the inflammatory phase (POD 0-2) (Table 1). Both administration of nBVL and d-galactose decreased wound size from POD 7 on (data not shown). In the healing period between proliferation and remodeling (POD 7–12), G-VI (nBVL) displayed greater CR and ER values than the other groups, though differences were not significant (Table 1).

### Histopathological Assessment

**Figure 2:**
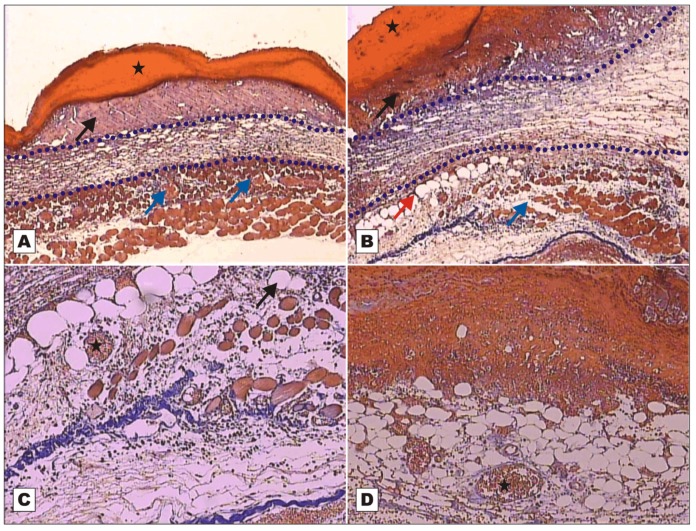
Photomicrographs of surgically induced skin wounds in mice treated with topical administration of BSA (Group III) and rBVL-1 (Group I); POD 2 (stained with Masson’s trichrome). **A**: BSA (Group III; control). See scab (star) covering the wound bed and intense inflammatory exudate (arrow), area of collagenolysis and reticular dermal edema under the exudate (between dashed blue lines), and subcutaneous skeletal muscle with congested vessels immediately below the dermis (blue arrows). Magnification: 4×; **B**: rBVL-1 (Group I). See thicker scab (star) over wound bed and less intense inflammatory exudate (arrow), area of collagenolysis, reticular dermal edema and intense inflammatory infiltrate under the exudate (between dashed blue lines), subcutaneous skeletal muscle with fibers separated by connective tissue immediately below the dermis (blue arrows), and fat cells near the area of collagenolysis (red arrow). Magnification: 4×; **C**: detail of subcutaneous skeletal muscle showing fat cells (arrow), congested vessels (star) and mild inflammatory infiltrate. Magnification: 10×; **D**: rBVL-1 (Group I). Congested vessels (star) bounded by collagen fibers embedded in proliferated unilocular adipose tissue below area of intense inflammatory exudate and scab.

Four samples of injured tissue were collected from each group on POD 2, 7 and 12 and submitted to histopathological evaluation. Wounds treated with BSA and rBVL-1 had scab sealing the opening in the epithelium (Figure 2).

Wounds treated with rBVL-1 presented mild inflammatory exudate, but the inflammatory infiltrate in the reticular dermis was intense (Figure 2B). Wounds treated with BSA had intense inflammatory exudate (Figure 2A). On the other hand, in wounds treated with rBVL-1, collagen fibers were seen around the congested vessels irrigating unilocular adipose tissue on POD 2, suggesting collagen-producing cell proliferation and, consequently, faster healing (Figure 2D).

**Figure 3:**
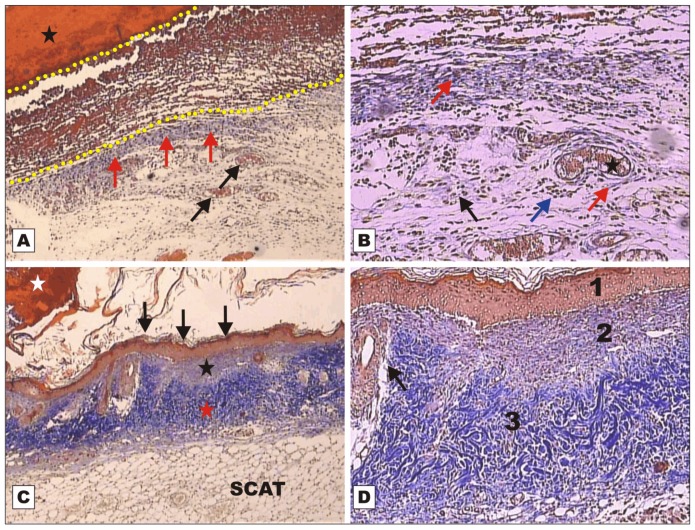
Photomicrographs of surgically induced skin wounds in mice treated with topical administration of BSA (Group III) and rBVL-1 (Group I); POD 7 (stained with Masson’s trichrome). **A**: BSA (Group III; control). Observe scab (star) covering the wound bed and intense inflammatory exudate (between dashed yellow lines), granulation tissue with congested vessels (arrow) and intense fibroblast proliferation below exudate, and blue band due to collagen synthesis (red arrows) immediately below the inflammatory exudate. Magnification 4×; **B**: BSA (Group III; control). Details of granulation tissue. Observe fibroblast proliferation (arrow) and mild inflammatory infiltrate around vessels (blue arrow), collagen band above granulation tissue (red arrow), and vessels in the granulation tissue (star). Magnification 10×; **C**: rBVL-1 (Group I). Scab detaching from epithelium (white star). Observe restructuring of the epithelial lining (arrow), dermis with areas of reduced subepithelial collagen deposition (star) and reticular dermis characterized by thickening of collagen fibers (red star). Observe restructured subcutaneous adipose tissue (SCAT) below the dermis. Magnification 4×; **D**: Detail of previous photo showing regular layers of restructured skin: 1) epidermis, 2) papillary dermis, and 3) reticular dermis, and budding skin appendages (arrow).

**Figure 4:**
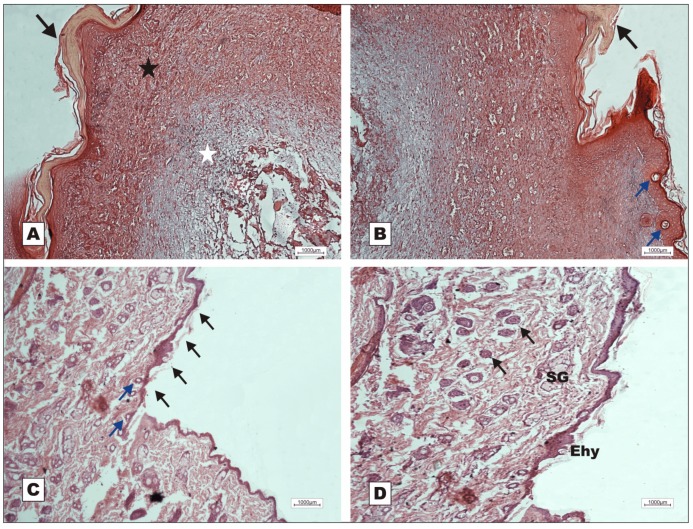
Photomicrographs of surgically induced skin wounds in mice treated with topical administration of d-galactose (Group IV) and rBVL-1 complexed with d-galactose (Group II); POD 12 (stained with Masson’s trichrome and hematoxylin-eosin). **A**: d-galactose (Group IV). Observe intact skin with newly formed luminal epithelium (black arrow), nearly closed wound and reduced keratin production, retracting granulation tissue just below the epithelium (star), indicating poor tissue reorganization and, further below, thickening of collagen fibers in the papillary dermis (white star). Magnification: 10×; **B**: d-galactose (Group IV). Presence of luminal epithelium (black arrow). Observe the epidermal hyperplasia with formation of budding skin appendage (blue arrows). Magnification 10×; **C**: rBVL-1 complexed with 2.3 mM d-galactose (Group II). Nearly closed wound with restructuring of the epithelial lining (black arrow). See the formation of budding skin appendage below the new epithelium (blue arrows). Magnification: 10×; **D**: rBVL-1 complexed with 2.3 mM d-galactose (Group II). Congested vessels are seen in the papillary dermis (arrows). See the epidermal hyperplasia (Ehy) and the presence of sebaceous glands (SG) as budding skin appendages. Magnification: 10×.

On POD 7, wounds treated with rBVL-1 presented restoration of the epithelial lining, subepithelial collagen deposition and thickening of collagen fibers in the reticular dermis with restructuring of the adipose tissue (Figure 3C). Figure 3D shows layers of restructured skin (epidermis, papillary and reticular dermis) and new skin appendages on animals treated with rBVL-1. This was not observed for animals treated with BSA (Figure 3A–B). In G-II (rBVL-1 complexed with d-galactose) and G-IV (d-galactose), tissue regeneration was observed only on POD 12 (Figure 4).

**Figure 5:**
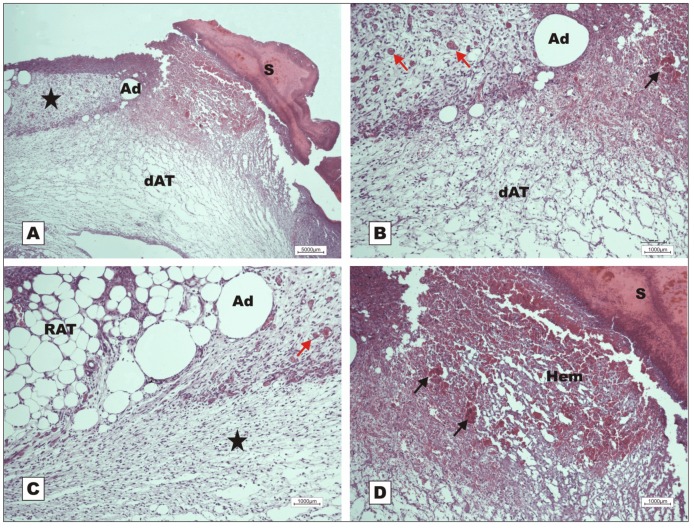
Photomicrographs of surgically induced skin wounds in mice treated with topical administration of nBVLcomplexed with 2.3 mM d-galactose (Group VII); POD 7 (stained with hematoxylin-eosin). **A**: Overview of damaged area. Observe scab sealing epithelial opening (S), early stage of granulation tissue formation (star) with intense acute inflammation and degenerating adipose tissue (dAT). Magnification: 4×; **B**: Detail of previous photo showing area with degenerating adipose tissue (dAT) and unaffected adipocytes (Ad) in the granulation tissue, vessels in granulation tissue (red arrows), and congested vessels (black arrow) in area with hemorrhage. Magnification: 10×; **C**: Granulation tissue originated from reactional adipose tissue (RAT). The red arrow shows congested vessels from granulation tissue. Observe area of intense inflammatory reaction (star). Magnification: 10×; **D**: Detail of open wound with scab, hemorrhage just below the scab, and congested vessels (arrows). Magnification: 10×.

**Figure 6:**
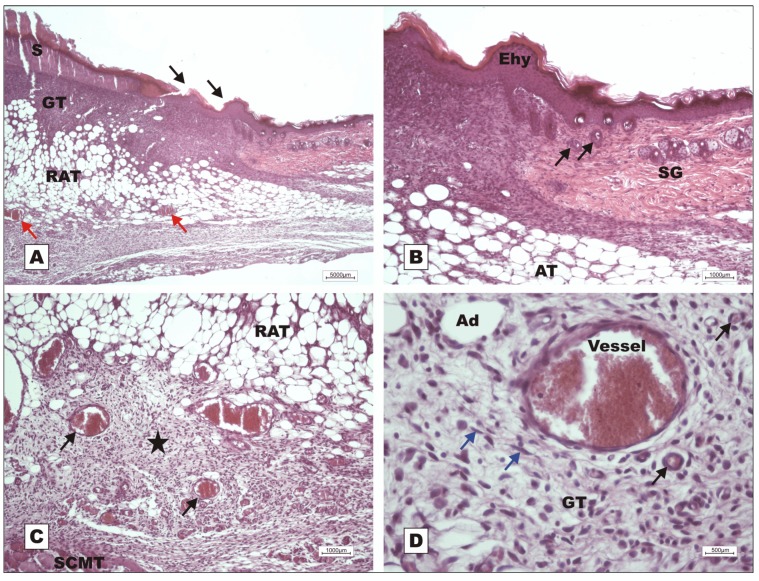
Photomicrographs of surgically induced skin wounds in mice treated with topical administration of nBVL (Group VI); POD 7 (stained with hematoxylin-eosin). **A**: Scab above granulation tissue originating from unilocular adipose tissue proliferating with active cell clusters (RAT) and congested vessels in granulation tissue (red arrows). Presence of recently formed epithelium on the board of scab area (black arrows). Magnification: 4×; **B**: Detail of previous photo showing epidermal hyperplasia (Ehy) on the border of scab area, budding skin appendages (black arrows) and sebaceous gland (SG). Observe the granulation tissue area along the adipose tissue (AT). Magnification: 10×; **C**: Detail of A showing area of active granulation tissue (star) with congested vessels (arrows) below the reactional adipose tissue (RAT). Observe subcutaneous muscle tissue (SCMT). Magnification: 10×; **D**: Detail of previous photo showing new vessels (black arrows) in the granulation tissue, indicating angiogenesis. Observe the presence of reactive fibroblasts on the granulation tissue (GT) area (blue arrows). Magnification: 40×

Healing was slow in G-VII (nBVL complexed with d-galactose), even during fibroplasia (POD 7). The animals in this group presented severe acute inflammation, little reaction of the adipose tissue to early formation of granulation tissue, hemorrhage, and degeneration of the fatty tissue below the dermis (Figure 5). On POD 7, development of epidermal hyperplasia for epithelial repair, active granulation tissue formation with adipose tissue proliferation and active cell clusters were observed in G-VI (nBVL) (Figure 6).

**Figure 7:**
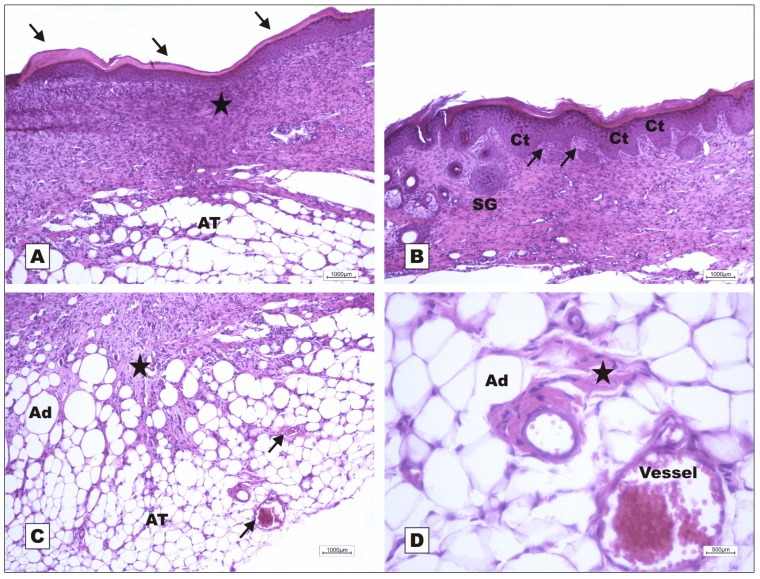
Photomicrographs of surgically induced skin wounds in mice treated with topical administration of nBVL (Group VI); POD 12 (stained with hematoxylin-eosin). **A**: Keratin deposition on the epithelial lining (arrows), thickening of subepithelial collagen fibers deposited in the dermis (star), and fat cells below the dermis (AT). Magnification: 10×; **B**: Detail showing the formation of epidermal ridges with consequent formation of dermal papillae (arrows), and newly formed skin appendages. Magnification: 10×; **C**: Detail showing bands of active collagen (star) moving towards the retracting adipose tissue, and congested vessels in granulation tissue (arrows) retracting along with the adipose tissue; **D**: Bands of active collagen in adipose tissue in retraction (black star). Remaining vessels of the granulation tissue can be noted. Magnification: 40×.

**Figure 8:**
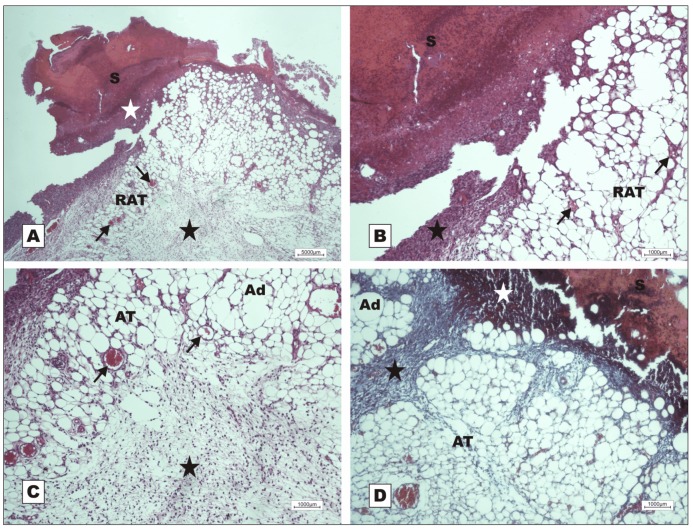
Photomicrographs of surgically induced skin wounds in mice treated with topical administration of 150 mM NaCl; POD 7 (stained with hematoxylin-eosin and Masson’s trichrome). **A**: Open wound with thick scab and dystrophic calcification (white star) sealing the epithelial opening (S). Observe early formation of granulation tissue originating from reactional adipose tissue (RAT) along with vessels (arrows), and waning inflammatory process (black star). Magnification: 4×; **B**: Granulation tissue with congested vessels in fat (arrows) immediately below calcified scab. Observe inflammatory exudates below scab (star). Magnification: 10×; **C**: Detail of A showing area of acute inflammation in slightly degenerated adipose tissue (star), and vessels of granulation tissue (arrows) in adipose tissue (AT) above the inflammation. Magnification: 10×; **D**: inflammatory exudate below scab (white star). Observe poor collagen deposition and granulation tissue in the dermis (black star) between areas of adipose tissue. Magnification: 10×.

On POD 12, the animals in G-VI (nBVL) presented restructuring of the epithelial lining with increased production of keratin, and dermal deposition of active collagen. Despite the inflammatory reaction (Figure 7A), bands of collagen were seen to proliferate towards the subcutaneous tissue, replacing the adipose tissue (Figure 7C), and ridges with dermal papillae and budding skin appendages were observed (Figure 7B). In G-V (saline), the healing process was delayed, as indicated by the presence of acute inflammatory response and early-stage granulation tissue on POD 7 (Figure 8).

The primary function of the skin is to serve as a protective barrier against the environment. The loss of skin integrity from injury or illness can lead to severe disability or even death [29]. Healing is a dynamic and well-organized biological process [30] involving cellular and biochemical components employed in the recovery of tissue morphology and function [31].

The development of technologies based on natural sources for the healing of skin wounds or ulcers is of major interest to researchers and other stakeholders in the biomedical field [32]. Of special importance are agents capable of enhancing tissue repair and reducing healing time [33,34].

Lectins are a structurally heterogeneous group of proteins with several direct biomedical and biotechnological applications [27]. Galactose-binding lectins are particularly useful because they interact with several endogenous molecules involved in innate and specific immune responses [13]. The ability of these macromolecules to activate immune cells, neutrophils, macrophages and mast cells [35,36] indicates a potential for accelerating wound healing and epithelial tissue regeneration [37].

In our study, wounds treated with nBVL presented stronger inflammatory signs characteristic of inflammatory response, inducing a proinflammatory response, than wounds treated with saline or BSA (controls). This appears to have contributed to the faster and more effective healing process observed in G-VI (nBVL).

Macrophages and mast cells are resident cells with surface glycoproteins and glycolipids which may act as ligands for lectins [13]. When activated, they release mediators capable of initiating inflammatory response [29,38]. Stimulation of adhesion molecules (VCAM-1, VE-Cadherin, ICAM-1, VCAM-1 and P-selectin) and chemotaxis (CXC receptors) triggers the migration of inflammatory cells to the damaged tissues [6,39]. Iordache and colleagues demonstrated that lectins from potatoes (*Solanum tuberosum*) and extracts of marigold (*Calendula officinalis*) are able to increase the proliferation of endothelial progenitor cells (EPCs) and modulate gene expression of adhesive molecules and chemotaxis, facilitating the attachment of the EPCs to the injured endothelium. Lopes and colleagues [40] demonstrated that plant lectins are capable of inducing histamine release by mast cells of different origins. In another study, lectins were shown to have a mitogenic effect on lymphocytes and to stimulate the production of proinflammatory cytokines such as IFN-γ and TNF-α, strongly activating macrophages cultured *in vitro* [41].

rBVL-1 (G-I) and nBVL (G-VI) induced faster healing than rBVL-1 complexed with d-galactose (G-II). These findings were confirmed by histopathological assessment (Figure 2, Figure 3, Figure 4, Figure 5, Figure 6, Figure 7 and Figure 8). The finding in G-I of regenerated skin layers already on POD 7 suggests rBVL-1 has a greater pro-healing potential than the other treatments tested in this study. The pro-healing effect was reversed when d-galactose blocked the CRD (carbohydrate recognition domain), suggesting healing was significantly improved in the presence of rBVL-1.

The lectin of *B. variegata* (nBVL) and its recombinant isoform (rBVL-1) appear to stimulate the mitogenic activity of resident cells, turning them into potent chemotactic agents for the recruitment of neutrophils through the release of cytokines, as observed in studies on other galactose-binding lectins such as *Vatairea macrocarpa* and *Artocarpus integrifolia* [13,17]. Mast cells are responsible for secreting large amounts of growth factors, including TGF-β and α [42]. TGF-β is a major growth factor involved in the regulation or stimulation of tissue repair, angiogenesis, granulation tissue formation, collagen synthesis by fibroblasts, and fibroplasia [42,43].

In a histological analysis of the effect of monosaccharides (hexoses) on wound healing in rats, Kössi and colleagues [44] found that treatment with d-galactose caused a considerable increase in the accumulation of granulation tissue. This may explain the pro-healing effect of d-galactose in relation to the controls.

Granulation tissue consists of new capillaries generated from pre-existing ones. The growth factors FGF2 and VEGF are responsible for the development of this tissue [45]. The formation of granulation tissue in the early postoperative period seems to suggest that nBVL and rBVL-1 induce the release of FGF2, VEGF and angiopoietins by endothelial cells modulated by cytokines produced by mast cells and macrophages.

Epithelialization starts within hours of tissue injury. In this process, the release of TGF-α and FGF flags are essential for the proliferation of epithelial cells and keratinocytes required for total wound closure. Moreover, fibroblasts are activated for collagen synthesis mediated by TGF-β released from mast cells and macrophages [43,46,47]. During tissue remodeling, the old collagen needs to be degraded by matrix metalloproteinases secreted mainly by macrophages and endothelial cells of the new vessels in the granulation tissue [48]. The new fibers deposited in the dermis (essentially the gradual replacement of type III collagen by type I collagen) are structurally more organized and will eventually form the new ECM [49].

According to Ishihara and colleagues, early healing is characterized by rapid epithelialization and wound contraction. Consequently, wound tensile strength increases, possibly due to greater deposition and stabilization of collagen fibers [50].

The CR and ER values observed in the present study strongly indicate nBVL and rBVL-1 stimulate the differentiation of fibroblasts into myofibroblasts, which is an extremely important event in the remodeling of connective tissue [51]. When fibroblasts are activated in damaged tissues, they differentiate into myofibroblasts which in turn exercise physical traction on the wound, stimulating contraction and helping remodel the collagen synthesized by fibroblasts during healing [43,51,52].

The difference in wound healing observed between animals treated with nBVL and rBVL-1 was most likely due to structural differences in the CRD of these lectins. rBVL-1 is but one of many potential isoforms of nBVL [23].

## 3. Experimental

### 3.1. Lectin Extraction and Purification

Lectin extracted from the seeds of *Bauhinia variegata* (nBVL) was purified by affinity chromatography on a lactose-agarose column (Sigma, St. Louis, MO, USA), then cloned and expressed in *E. coli* to produce the recombinant isoform (rBVL-1), following the method described by Pinto and coworkers [23].

### 3.2. Induction and Treatment of Skin Wounds

The experimental protocol was approved by the Ethics Committee of Ceará State University (UECE) under entry #11042434-4 and all animals were treated following the recommendations of the Brazilian College of Animal Experimentation (COBEA) and the Guide for the Care and Use of Laboratory Animals of the US Department of Health and Human Services (NIH publication No. 85–23, revised 1985).

The *in vivo* study employed eighty-four 10-week-old male Swiss albino mice (*Mus musculus*) weighing 35.0 ± 5.0 g, supplied by the laboratory animal facility of the Federal University of Ceará (BIOCEN/UFC). During the experimental procedures, the animals were kept in individual cages in a controlled environment (circadian cycle, 25 ± 2 °C, 55 ± 10% humidity, food and water *ad libitum*) at the School of Medicine in Sobral (UFC).

Prior to the surgical procedure, the animals were randomly distributed into seven groups (n = 12) according to the topical treatment administered: G-I (200 µg/mL rBVL-1); G-II (200 µg/mL rBVL-1 complexed with 2.3 mM d-galactose); G-III (200 µg/mL bovine serum albumin); G-IV (2.3 mM d-galactose); G-V (150 mMNaCl); G-VI (200 µg/mL nBVL); G-VII (200 µg/mL nBVL complexed with 2.3 mM d-galactose).

The mice were then anesthetized with subcutaneous administration of 2% xylazine with 10% ketamine hydrochloride (10 mg/kg and 115 mg/kg, respectively) [53], followed by trichotomy and antisepsis of the dorsal thoracic region with povidone-iodine and sterile saline solution (150 mM NaCl). After marking the skin with a sterile mold (1.00 cm^2^), circular aseptic skin wounds were created by incision with a scalpel (#15) followed by resection of the subcutaneous tissue with fine dissection tweezers. Immediately after the surgical procedure, the wounds were treated topically for 12 days according to treatment group. Animals treated with lectins and BSA received a single daily dose of 20 µg/100 µL; the remainder received 100 µL.

### 3.3. Evaluation of Healing Potential

Wounds were measured daily for 12 days in order to evaluate the healing potential of each treatment. The wound area was expressed as mean ± standard deviation, as previously reported [54]. The wound contraction rate (CR) was expressed as change in wound area (mm^2^) over time (day), according to the following equation:

CR = ∆ area ÷ ∆ time (day)

The epithelialization rate was expressed as growth of new epithelium (mm^2^) over time (day), according to the following equation:

2ER = ∆ area ÷ ∆ time (day)

The animals were anesthetized prior to the collection of histopathological material and subsequently euthanized [54]. Samples (4 per group) of injured tissue were collected on the 2nd, 7th and 12th postoperative day (POD), fixed in 10% formaldehyde (v/v) buffered in 0.01 M PBS (pH 7.2), prepared in 5-mm cuts for routine histological analysis [55] and stained with hematoxylin-eosin (HE) and Masson’s trichrome. The histopathological assessment included the following parameters: Presence of scabs, re-epithelialization, collagen deposition, neovascularization and exudate. The analysis was performed under a Leica light microscope (model DM 500) at 4, 10 and 40× magnification.

### 3.4. Statistical Assessment

Intergroup differences in CR and ER values were analyzed with one-way ANOVA followed by the Tukey post-test. Differences between wound area and closure percentage were tested with the Mann-Whitney test. The data were processed with the statistics software GraphPad Prism v.3.00 for Windows^®^. Values are given as mean ± standard deviation. The level of statistical significance was set at *p* < 0.05.

## 4. Conclusions

Our results indicate that lectin extracted from the seeds of *Bauhinia variegata* (nBVL) can stimulate the healing process of skin wounds in mice, possibly by acting on cells of the immune system, enhancing proinflammatory response, collagen synthesis by fibroblasts and angiogenesis by modulating the release of inflammatory cytokines and growth factors. The recombinant isoform of the lectin (rBVL-1) displayed pro-healing effects as well.

The secretion of growth factors and consequent phenotypic change of fibroblasts into myofibroblasts may explain the observed acceleration in wound closure (faster wound contraction and increased collagen deposition by fibroblasts, even in the inflammatory phase), with effective healing accomplished by POD 7.

## Figures and Tables

**Table 1:** Contraction rate (CR) and epithelialization rate (ER) of surgically induced skin wounds in mice treated with rBVL-1 (Group I), rBVL-1 complexed with d-galactose (Group II), bovine serum albumin (Group III), d-galactose (Group IV), saline (Group V), nBVL (Group VI) or nBVL complexed with d-galactose (Group VII). Figures are mean values ± standard deviation.

POD	0–2		2–7		7–12	
Parameter	CR	ER	CR	ER	CR	ER
**Group I**	7.708 ± 1.345 *	3.854 ± 0.672 *	3.800 ± 0.704	1.900 ± 0.352	48.50 ± 24.13	24.25 ± 12.07
**Group II**	0.833 ± 0.441	0.416 ± 0.220	5.275 ± 0.915	2.638 ± 0.457	40.50 ± 9.35	20.25 ± 4.67
**Group III**	3.667 ± 1.008	1.833 ± 0.504	5.675 ± 0.630	2.838 ± 0.315	35.50 ± 3.61	17.75 ± 1.80
**Group IV**	2.542 ± 1.532	1.271 ± 0.766	4.775 ± 1.113	2.388 ± 0.556	69.70 ± 25.37	34.85 ± 12.69
**Group V**	2.250 ± 1.689	1.125 ± 0.845	4.625 ± 1.375	1.542 ± 0.555	14.75 ± 1.80	2.458 ± 1.08
**Group VI**	3.958 ± 1.345	1.979 ± 0.672	5.925 ± 0.865	2.963 ± 0.433	61.00 ± 9.33	30.50 ± 4.66
**Group VII**	2.542 ± 1.167	1.271 ± 0.583	6.125 ± 1.616	3.063 ± 0.808	52.75 ± 1.93	26.38 ± 0.96

POD = postoperative day. Parameter units: mm^2^/day. * Statistically significant when compared to Groups II, IV, V and VII (*p* < 0.05; ANOVA and post-Tukey test).
